# The Impact of Nano-Al_2_O_3_ on the Physical and Strength Properties as Well as on the Morphology of Cement Composite Crack Surfaces in the Early and Later Maturation Age

**DOI:** 10.3390/ma14164441

**Published:** 2021-08-08

**Authors:** Wioleta Iskra-Kozak, Janusz Konkol

**Affiliations:** Faculty of Civil and Environmental Engineering and Architecture, Rzeszow University of Technology, Poznanska 2, 35084 Rzeszow, Poland; jk7@prz.edu.pl

**Keywords:** nanoparticles, nano-alumina, compressive strength, fractal dimension

## Abstract

This article presents the effect of aluminum nanoxide on the physical, strength and structural properties of cement mortars. The mortars were made with a water to binder ratio of 0.5 and a binder to sand ratio of 1:3; and 1%, 2%, 3% and 4% of aluminum nanoxide, respectively, were used by cement weight. First, the consistency of nano-Al_2_O_3_ mortars was tested. Next, after 7 days of sample maturation, compressive and flexural strength tests were carried out and continued after 28 and 90 days of the maturing of the mortars. The best test results were obtained for mortars with the addition of 1% aluminum nanoxide, the compressive strength of which increased by about 20% compared to the reference mortars. The water absorption and rising capillary tests as well as SEM observations were also performed. Another aim of the article is the analysis of the fracture morphology of nano-Al_2_O_3_ modified mortars. It is assumed that a change of the microstructure of the hardened cement paste affects not only the properties of the modified mortars but also the roughness of the fractures formed as a result of the destruction of the surface. Roughness analysis was performed with methods and tools relevant to fractal geometry. The fractographic analysis showed a significant influence of the modifier in the form of nano-Al_2_O_3_ on the values of fractal dimensions. The lowest values of the fractal dimension *D* and the fractal dimension of the *D_RP_* roughness profile of the fracture surface profile lines were obtained for nano-Al_2_O_3_ modified mortars. The conducted research proved the fractal dimension to be a parameter extremely sensitive to modifications of mortar composition as well as changes related to the maturation time.

## 1. Introduction

Nanotechnology plays an important role in various areas of our life, especially in the science of concrete [1,2]. Currently, nanotechnology is developing rapidly in various industries. The innovation of nanotechnology lies in the fact that the morphology and size of individual elements of a nanostructure may have a greater impact on the properties of the material than its chemical composition. New properties are revealed when the critical grain size is exceeded, which is called “the nanoscale effect”. Some scientists include the cement industry among the top ten potential beneficiaries of nanotechnology development in the near future. The use of nanoparticles in cement composites produces concrete with characteristics such as high strength, water-permeability or resistance to acids and high temperature [3,4,5,6,7]. The most commonly used nanoproducts in cement technology include nano-SiO_2_ [8,9,10,11,12,13,14,15,16,17] and nano-TiO_2_ [18,19,20]. Most of the research is carried out with the use of carbon nanomaterials in cement composites, mainly nanotubes and graphene [3]. Recent work has been published on the use of nano-Al_2_O_3_ and nano-Fe_3_O_4_ in cement technology [21].

Aluminum nanoxide fills the voids between cement grains, as a result of which capillary pores are closed [22,23,24]. Well-dispersed nanoparticles accelerate cement slurry hydration and contribute to the formation of small crystals and small homogeneous clusters in the C–S–H phase. Moreover, nanoparticles are involved in pozzolanic reactions or accelerate them, which causes wear in the hardening cement of chemically unstable Ca(OH)_2_ portlandite and the formation of an additional amount of C–A–S–H phase [24]. In addition, nanoparticles improve the contact structure in the interface between cement grains, which results in a stronger bond and a reduction in the number of possible cracks. Aluminum anoxide is another nano-sized material used in cement-based materials, which has a beneficial effect on the microstructure (pore structure and transition zone) [23,24], increases the modulus of elasticity [23] and improves compressive and bending strength [23,25] and also increases resistance to high and low temperatures [26,27,28]. Behfarnia K. and Salemi N. [28] confirmed that the frost resistance of concrete containing nano-Al_2_O_3_ is higher than that of concrete containing the same amount of nano-SiO_2_. The chloride binding capacity can be improved by introducing nano-Al_2_O_3_ into cement paste. The bound chloride content is increased by 37.2% at 0.05 mol/L NaCl solution with 5.0% nano-Al_2_O_3_ [29]. The effect of nano-Al_2_O_3_ on the mechanical properties was generally attributed to its physical action (filler effect), which led to accelerated hydration in the early period [25,30]. Some studies have shown that nanoparticles have no effect [31] or only a slight effect on the increase of strength [23,32], possibly due to the dispersion and agglomeration of nanoparticles in cement-based materials. In the early stage of the hydration process, the physical effect of nano-aluminum oxide has been well demonstrated in the literature, while the chemical effect of aluminum nanoxide on the early stage of hydration is being examined. The isothermal calorimeter-based evidence showed that nano-alumina accelerated the reaction of tricalcium aluminate (C_3_A) by increasing the second peak of the heat flow curve, indicating possible dissolution of nano-aluminum particles in the pore solution [25]. As an amphoteric oxide, nano-alumina is practically insoluble in water in ambient conditions, while its solubility increases significantly in acidic or alkaline solutions. Roelofs [33] found that nano-Al_2_O_3_ (γ phase) quickly dissolves on contact with an aqueous solution at pH = 11, and the actual aluminum concentration increases rapidly until it reaches its maximum value (at 25 °C, 2.5 mmol/L). It is commonly known that the pH of a pore solution in a cementitious binder can reach a very high level within minutes after being mixed with water. The high alkalinity of the pore solution (pH > 12.5) would have a significant effect on the solubility of nano-aluminum oxide, chemically influencing the aluminate reaction during the early phase of hydration.

Following the few published experimental works and their original research, the authors of this study focused on the analysis of the morphology of fractures of nano-Al_2_O_3_ modified mortars. Fracture morphology analyses provide a quantitative description of the structure of materials, including cementitious matrix materials. Importantly, they provide information on the image of damage which can be described in a simple way by means of fractal geometry [34,35,36]. In the approach specific for materials engineering, the results thus obtained may be a basis for the search for functional relationships which, apart from relating these parameters with other properties of materials and their application in design [37], may also provide a basis for modelling, including the phenomenon of cracking [38,39,40,41,42]. The extensive fractographic analysis presented in this article confirms the feasibility of using this type of approach. Due to significant scatters in fractographic parameters, it was necessary be carry out this extensive statistical analysis. The presented research will significantly supplement the knowledge in the field of modification of cement composites with aluminum nanoxide.

## 2. Materials and Methods

### 2.1. Materials Used

− Cement: CEM I 42.5 R. The physical and mechanical properties of Portland cement used following the requirements of PN-EN 197-1: 2012 (Table 1 and Table 2).
− Aggregate: Quartz sand, grain size up to 2 mm. Sands used followed the requirements of PN-EN 12620:2004. Producer: ZEK STRZEGOCICE II (Table 3).
− Superplasticizer: Masterglenium 115—superplasticizer based on polycarboxyl ethers.
− Aluminum nanoxide: nanoparticles whose grain size declared by the manufacturer is 40 nm (Table 4)

**Figure 1:**
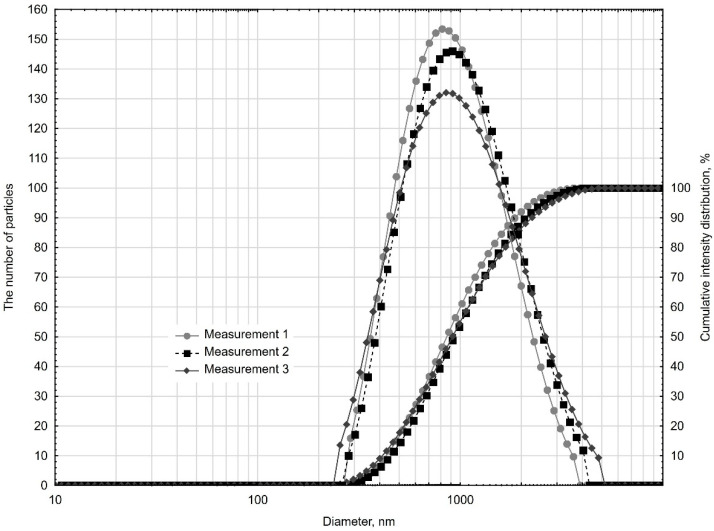
Cumulative curves and normalized intensity distribution curves.

To determine the size of nanoadditive particle, a NanoPlus HD wet dispersion system was used to measure particle sizes from 0.1 nm to 12.300 nm (conforming to ISO13321 standard for particle size). The dispersing liquid was water. Figure 1 presents the results of the three samples. The results are displayed graphically as cumulative curves and normalized intensity distribution curves. The analysis was also performed with the use of methanol as a dispersant. The obtained results were similar and did not confirm the particle size declared by the manufacturer.

A unimodal distribution of the curve of the normalized intensity distribution was obtained, and the repeatability of the results for three samples confirmed the homogeneity of this material. The occurrence of grains with diameters ranging from about 250 nm to 5000 nm was found. The mean chord of three measurements was 856.4 nm, with 90% occurrence of grains of the diameter below 2072.4 nm. The reason why we checked the particle size distribution was because of the results of preliminary studies and SEM observations.

### 2.2. Experimental Methods

The scope of the experimental methods included the following:

− Testing the consistency of mortars employing the spreading table method after PN-EN 1015-3:2000 [43]. The measurement consisted of determining the sample flow diameter spread on the flow table. The diameter of the flow was measured in millimeters.
− Flexural and compressive strength of testing mortars after 7, 28 and 90 days of mortar curing after PN-EN 1015-11:2001 [44]. The determination of flexural strength of mortars was carried out on three samples with dimensions of 40 × 40 × 160 mm^3^ using a testing machine. Compressive strength was determined on the halves of the three samples that remained after the mortar bending test.
− Testing water absorption resulting from the capillary rise after 28 days of mortar curing after PN-EN 1015-18:2002 [45]. During the test, the moisture level of the samples that were placed in water from the water level in the vessel was determined, as well as the increase in the weight of the sample. The measurement was carried out on six halves of samples.
− Absorption testing of mortars after 28 days of mortar curing after PN-B-04500:1985 [46]. During the test, the water mass that can be absorbed by a sample immersed in water under atmospheric pressure was determined. The measurement was carried out on three samples.
− Analysis of the morphology of the fractured (in a flexural strength test) surfaces of the mortar sample fractures was performed using a Taylor Hobson Talysurf CLI 1000 laser profilometer (Taylor Hobson Ltd., Leicester, UK) with software and the “FRAKTAL_Dimension2D” program (J. Konkol, FRACTAL_Dimension2D, a program, 2000, Poland ) [47]. The surfaces were separated out by 20 profile lines of 30 mm each. The number of profile lines was selected based on the research described in [48]. The measurements were performed with a discretization step of 1 µm, resulting in 30,001 points describing the profile line. The fractal dimension D was determined by the enclosing boxes method and the total profile height of the Pt profile. The analysis was carried out in two ways, i.e., for the profile line, and for the roughness profile separated out from this line. This approach was aimed at finding a solution that is more sensitive to changes in the microstructure of hardened cement paste due to its modification with aluminum nanoxide. SEM studies of the microstructure of the hardened cement slurry performed with a JEOL 5500 LV scanning microscope (Joel, Peabody, MA, USA) were carried out. The observations were run on randomly selected fracture surfaces of the samples used in the fracture resistance tests. Samples for SEM tests, after being stuck to the table, were sputtered with gold.

### 2.3. Proportions of Cement Mortars

The mixtures were divided into five groups on the basis of the variable amount of introduced aluminum nanoxide (0%, 1%, 2%, 3%, 4%). The amount of nanoparticles was added based on the weight of the cement. Alumina nanoparticles in the form of powder were added to the mixing water in order to better disperse the nanoparticles, and they were dispersed using a Sonopuls HD 3200 sonicator (amplitude 70%, mixing time 15 min) [49].

Masterglenium 115 superplasticizer was added in proportion to the weight of the binder, the amount of which increased proportionally to the amount of aluminum nanoxide added. It acted as a dispersant. A constant water to binder ratio of 0.5 was adopted. Designations and compositions of individual mixtures are presented in Table 5.

## 3. Results and Discussions

### 3.1. Consistency

The consistency of the mixture containing aluminum nanoxide introduced as a partial cement replacement depends on the amount of this additive. The aim of the research was to obtain a constant consistency with a water to binder ratio. Aluminum nanoxide impacted the consistency of the tested mortars. The workability of nano-Al_2_O_3_ modified mortars deteriorated with the increase in the amount of introduced nano-alumina. The water demand of the binder increased with the increase in the amount of aluminum nanoxide. In order to maintain a solid consistency, a superplasticizer was used, which, in addition to the function of a dispersing agent, also acted as a liquefier. Analysis of the results of all the tested mortars indicated that they are comparable. The spread of the tested mixes oscillated within the range of 14.5–16 cm. All the tested mixtures were workable.

**Figure 2:**
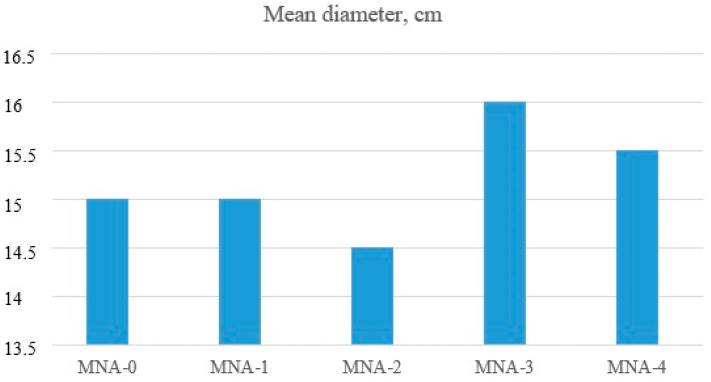
Mortar consistencies tested with the flow table test.

The consistency of all mortars is shown in Figure 2.

### 3.2. Flexural Strength and Compressive Strength

In order to evaluate the influence of aluminum nanoxide on the strength properties of cement mortars, three samples of each series with the dimensions of 40 mm × 40 mm × 160 mm were made and cured at the temperature of 20 °C ± 2 °C and at the relative humidity of 65% ± 5%. The flexural strength and compressive strength of the mortars were determined after 7, 28 and 90 days of curing. The tests were carried out on an automatic press for measuring the strength of cement samples after PN-EN 196-1. The obtained results are shown in Table 6 together with a standard error of the mean value.

The analysis of the obtained flexural strength results indicated that the results for all the series of the tested mortars are comparable after 7, 28 and 90 days of curing of the tested mortars. It should be noted that as the amount of introduced alumina nanoparticles increases, the flexural strength decreases. Significant differences in the results can be seen in the compressive strength test of the mortars. Aluminum nanoxide had a positive effect on the early compressive strength of mortars, but also on the compressive strength of mortars after 28 and 90 days of curing compared to the reference mortar samples. The increase in compressive strength results from the rapid consumption of Ca(OH)_2_ during the hydration of Portland cement, especially in the initial period associated with the high reactivity of nano-Al_2_O_3_ particles. The presence of Al_2_O_3_ nanoparticles in the cementing system not only improves the particle packing density but also consumes portlandite (Ca(OH)_2_). The portlandite consumption is caused by the pozzolanic reaction, forming additional C–S–(A)–H gel that fills the capillaries and reduces the pore structure of concrete and consequently, increases its strength. Nano Al_2_O_3_ particles act as nuclei for the cement paste further accelerating the cement hydration reactions. Due to their high reactivity, and as a nano-filler material, nano particles lead to the densification of the cement microstructure and, hence, reduced porosity and enhanced strength [50,51,52]. The highest compressive strength of the nano-Al_2_O_3_ modified mortars was demonstrated by the mortar with the addition of 1% alumina nanoparticles. Compared with the reference mortar MNA-0, MNA-1 mortar showed an increase in compressive strength after 7 days by about 20%. With the increase of the addition of aluminum nanoxide, the compressive strength of mortars decreased, but it was still higher than that of reference mortars. This was most likely caused by the uneven distribution of aluminum nanoxide in the mortar.

**Figure 3:**
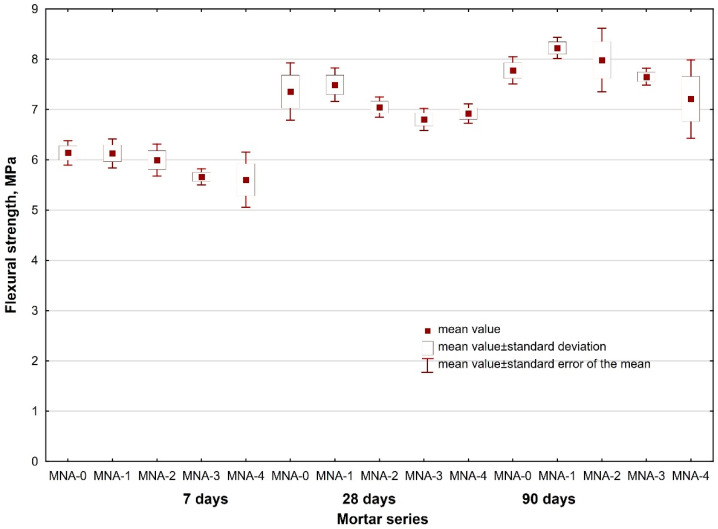
Scatter of results of flexural strength vs. nano-Al_2_O_3_ proportion and mortar age.

**Figure 4:**
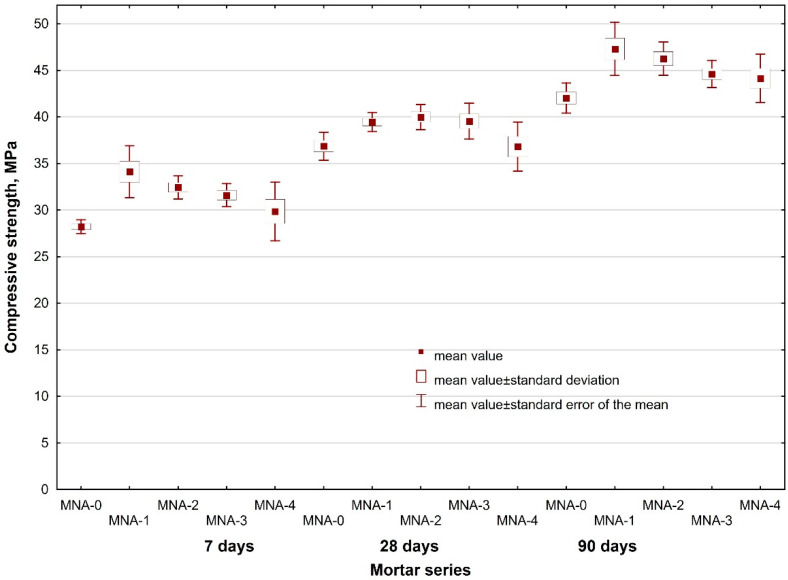
Scatter of results of compressive strength vs. nano-Al_2_O_3_ i proportion and mortar age.

Additional in-depth statistical analyses with STATISTICA (v. 13.1, TIBCO Software Inc., Palo Alto, CA, USA) of the results of tests on mortars’ flexural and compressive strengths, taking into account the proportion of Al_2_O_3_ and the mortars’ age (15 series of mortars in total) were performed. An F Snedecor (Fisher) qualitative analysis was done in order to demonstrate the impact of the adopted variables on the property tested. The analysis of the results of tests of both flexural strength (test statistic F = 14.27) and compressive strength of mortars (test statistic F = 54.93) yielded critical levels of significance *p* close to 0, which proves the statistically significant effects of the adopted independent variables on these output values. At the same time the F test is a test of the equality of mean values of flexural/compressive strengths, and its results confirmed the statistical differentiation of the mean values in particular series depending on the adopted variables. The scatter of the results of flexural and compressive strengths in particular mortar series is shown graphically in Figure 3 and Figure 4.

To detect any potential impact of other variables on the parameters tested, the homogeneity of variance was checked. The factors that might make the scatter wider included a technological issue of adequate dispersion of nano-Al_2_O_3_ in the mortar. The homogeneity of variance was checked with the Brown–Forsythe test. For both parameters the critical level of significance *p*, which was 0.97 for flexural strength and 0.53 for compressive strength, indicated variance homogeneity. The fact that homogeneity of variance was achieved confirms adequate dispersion of nano-Al_2_O_3_W in the mortar.

Through the application of the multiple regression method together with the adoption of nano-Al_2_O_3_ proportion and mortar age as independent variables and flexural strength *f_r_* or compressive strength *f_c_* of mortars as a dependent variable, the following dependences, (1) and (2), were obtained:

(1) $$f_{r}=6.43+0.02\cdot T-0.32\cdot x,\;N=\;45,\;R=\;0.83,\;p=\;0.000\;$$

(2) $$f_{c}=31.96+0.15\cdot T,\;N=50,\;R=0.86,\;p=0.000$$

where *T* is the age of mortar, *x* is the proportion of nano-Al_2_O_3_ and *N* is the number of cases.

The statistical significance of correlation was confirmed (*p* close to 0). For model (2) of multiple regression, only the insignificance of the term for the linear effect of the variable of nano-Al_2_O_3_ proportion was shown. For model (1), based on the results of multiple regression analysis, it was found that the contribution of the variable of mortar age in the prediction of flexural strength was about three times greater than the contribution of nano-Al_2_O_3_ (regression standardized coefficients ratio of 0.79/0.26).

Because of low values of correlation coefficients in the case of multiple regression, for the search for dependencies, a non-linear estimation method adopting the output model variables (mortar age (*T*), nano-Al_2_O_3_ proportion (*x*)), also in the second power, was employed. After the elimination of coefficients, statistically insignificant functional dependences were reached:

(3) $$f_{r}=5.63+0.078\cdot T-0.00057\cdot T^{2}-0.163\cdot x^{2},R=0.92$$

(4) $$f_{r}=26.27+0.450\cdot T+7.418\cdot x-0.003\cdot T^{2}-3.635\cdot x^{2},R=0.94$$

**Figure 5:**
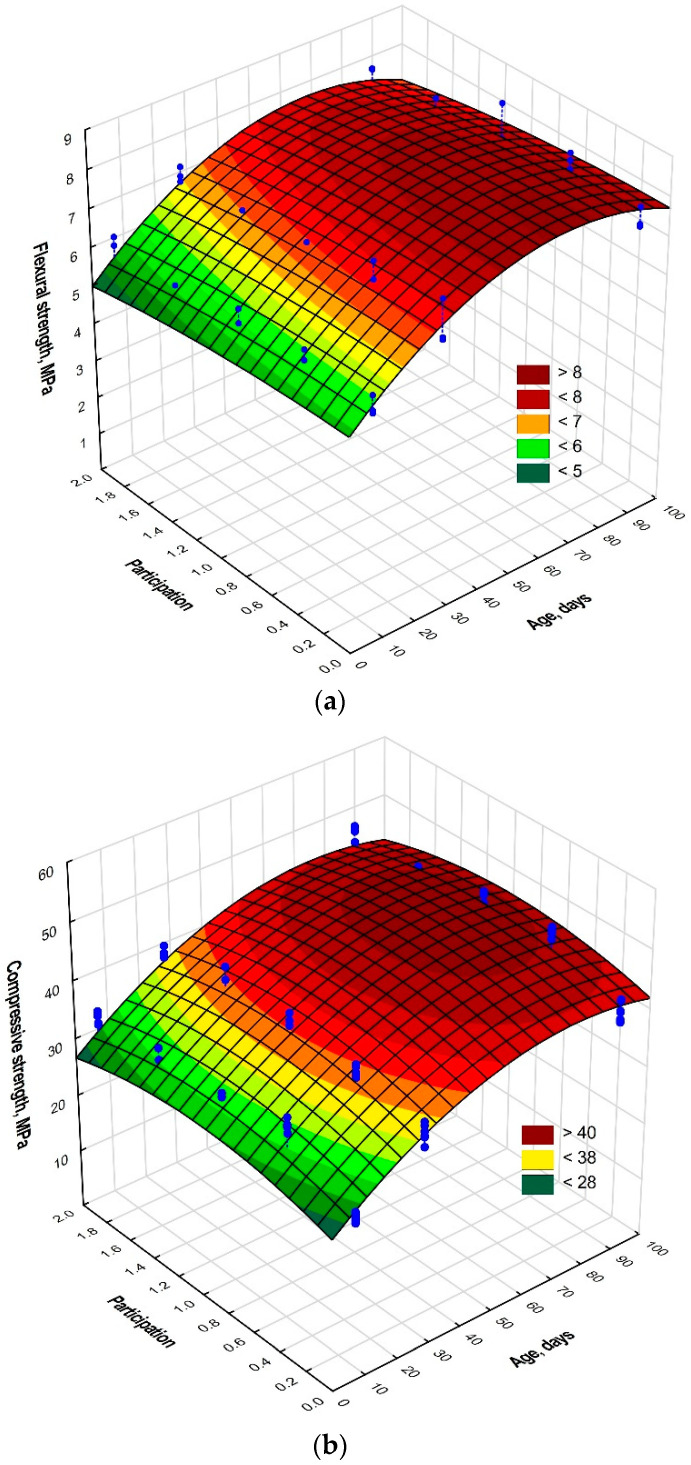
Approximating functions for (**a**) mortar flexural strength, (**b**) mortar compressive strength.

The proposed functions (3) and (4) were valid only in the experiment, i.e., for mortars aged seven to ninety days. The maximal and minimal error of +3.0% and −3.3%, respectively, of the estimation of mean value expected on the basis of Equation (3) compared with the observed (real) value was obtained. The mean absolute error of the estimation of this value was 1.7%. In the case of compressive strength and the use of Equation (4), the maximal and minimal error was +6.0 and −3.7%, respectively, and the mean absolute error of the estimation of mean compressive strength was 1.8%. Better fitting of the results of experimental studies to the model (increase of correlation coefficient *R*) was observed. The surface diagrams thereof are shown in Figure 5.

In the case of mortar flexural strength (Figure 5b), its decrease with an increase of nano-Al_2_O_3_ proportion was conspicuous in mortars tested after 7, 28 and 90 days of curing. With mortar age, its flexural strength increased. On the other hand, in the diagram of compressive strength vs. mortar age and nano-Al_2_O_3_ proportion, the maximum was noticeable at a nano-Al_2_O_3_ proportion of about 1%. In this case, an increased compressive strength of mortar with age was also observed.

### 3.3. Capillary Water Absorption

**Figure 6:**
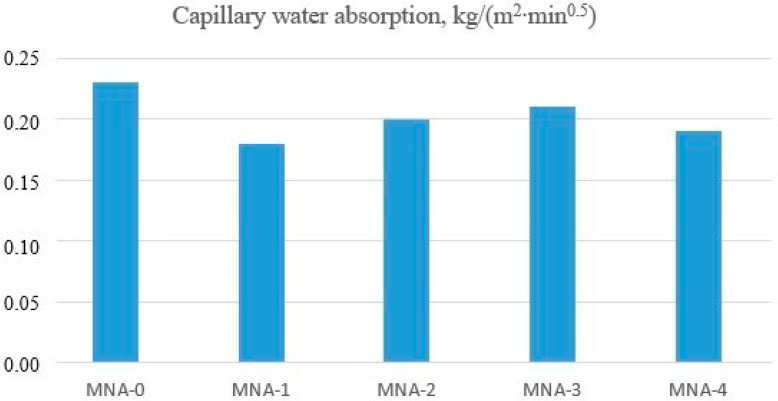
Capillary water absorption.

Determination of capillary rise of water was carried out after 28 days of curing on six halves of samples from each series of tested mortars. The level of moisture in the samples, which were set in water up to the level of water in the vessel, was determined, as well as was their weight gain. The obtained results are shown in Figure 6.

The analysis of the obtained results indicated that the lowest capillary adsorption coefficients were obtained for mortars modified with aluminum oxide nanoparticles. Compared with the control mortar, they were lower by about 15%. The lowest capillary adsorption coefficient was shown by MNA-1 mortar with 1% content of aluminum nanoxide. Compared with the reference mortar, the capillary adsorption coefficient of MNA-1 mortar was lower by 21%. The lower capillarity of the nano-Al_2_O_3_ modified mortars was most likely caused by filling the capillary pores with aluminum nanoxide particles. Aluminum nanoxide acts as a “nano-filler” and thus improves the microstructure of cement composites. In the analysis of the capillarity of only the mortars modified with aluminum oxide nanoparticles, it was observed that with a higher content of nano-Al_2_O_3_, the mortars showed an increase in the value of the capillary adsorption coefficient by about 10%. This was probably caused by uneven distribution of aluminum nanoxide in the mortar, so that this additive did not fill most of the capillary pores contained in the tested mortars. When a larger amount of nano-aluminum oxide was added to the mortar, homogenization was hindered. One of the characteristics of aluminum nanoxide is a large specific surface area, which causes the formation of nano-Al_2_O_3_ agglomerates and thus weakens the structure of the resulting cement paste. Unlike in the research by Oltulu and Şahin [53], in our study the addition of nano-aluminum to the mortars gave the following results: mortars with the addition of nano-Al_2_O_3_ showed an increase in capillarity compared with the control group, 3% for F0.5NA, 4% for F1.25NA and 10% for F2.5NA. The addition of nano-Al_2_O_3_ powders in proportions of 0.5% and 2.5% increased the coefficient of capillarity by 4% and 1%, respectively.

### 3.4. Water Absorption

**Figure 7:**
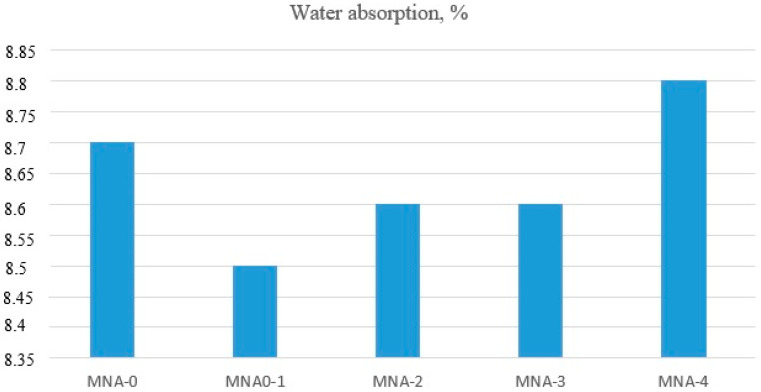
Water absorption.

The mortar water absorption tests were conducted after 28 days of curing on three samples of dimensions of 40 mm × 40 mm × 160 mm from each series of tested mortars. The water mass that could be absorbed by a sample immersed in water under the influence of atmospheric pressure was determined. The test results are shown in Figure 7.

The results showed a slight influence of the addition of aluminum oxide nanoparticles on the absorbability of the tested cement mortars. All the results were comparable and oscillated around 8.6%. The analysis of the results of only nano-Al_2_O_3_ modified mortars indicated an increase in water absorption along with an increase in the amount of aluminum oxide nanoparticles addition. The higher the amount of added nano-Al_2_O_3_ the more difficult it was to evenly distribute it in the mortar, which probably affected the results of the absorption test. Raje Gowda et al. [54] observed that the addition of nano-Al_2_O_3_ did not show any major differences between the modified mortar and the reference sample. The mean values were 8.37%, 7.80%, 8.17% and 8.50% for 0%, 1%, 3% and 5%, respectively. They assumed that when the nano-aluminum concentrations were low, the pores and smaller voids in the mortars were not completely filled and therefore showed increased absorption. On the other hand, at 5% nano-Al_2_O_3_, the agglomeration resulted in nonuniform distribution of the nano-Al_2_O_3_ in the mortars that left the pores unfilled. These unfilled pores in mortars again helped in increasing the absorption of water.

### 3.5. Morphological Analysis

**Figure 8:**
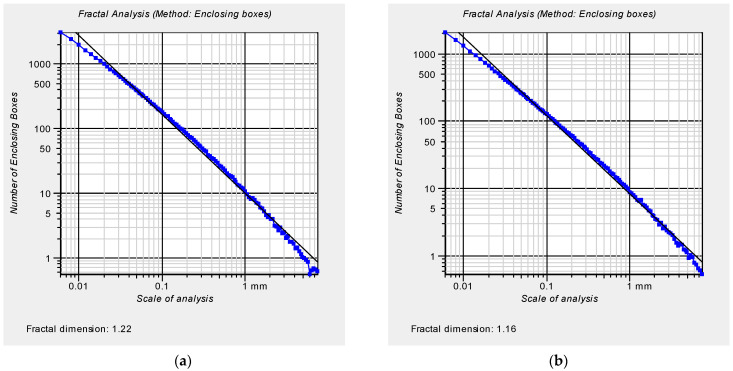
The result of the fractal analysis of an example and representative profile line separated out from the fracture surface of the mortar unmodified with aluminum nano-oxide after 7 days of maturation (**a**) and with 1% addition of nano-aluminum oxide (**b**).

The morphology of the mortar sample fracture surfaces formed due to destruction was analyzed after 7, 28 and 90 days of mortar curing. The fracture surface was selected from the samples used in testing the flexural strength of these mortars. The fractal dimension D was chosen as the basic parameter describing the morphology of the fracture surface. The fractal dimension was determined using the enclosing the boxes method. The analysis was performed as a 2D issue by extracting profile lines from the fracture surfaces. On the surfaces, 20 profile lines of 30 mm each were separated out. Measurements were taken with a discretization step of 1 µm, which provided 30,001 points describing the profile line. For each line, the value of the fractal dimension was determined (Figure 8).

**Figure 9:**
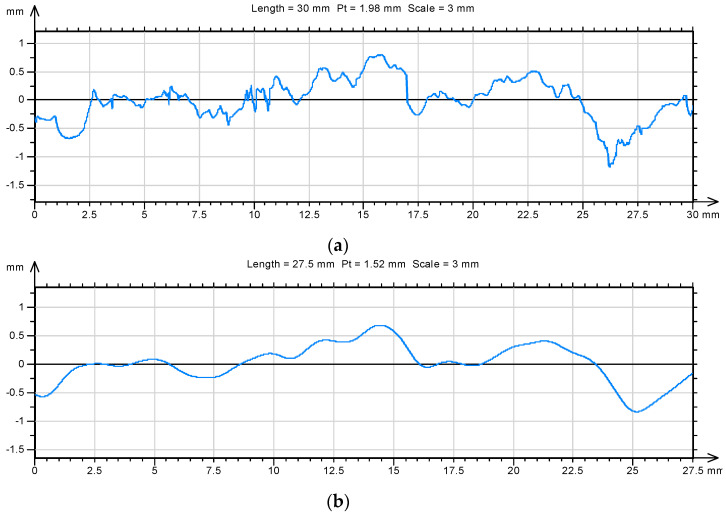

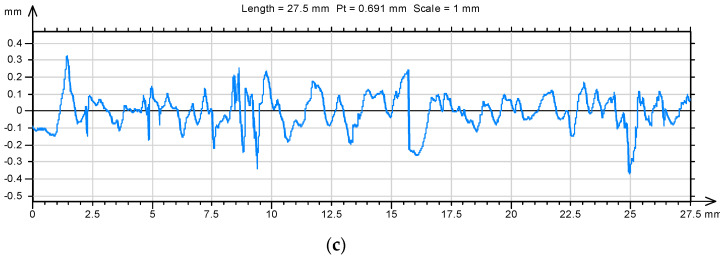
An example of a profile line (**a**) separated out on the surface of a mortar sample after 7 days of maturing with aluminum nanoxide (*D* = 1.22) and its waviness profile (**b**) and roughness profile (**c**) (*D* = 1.21).

**Figure 10:**
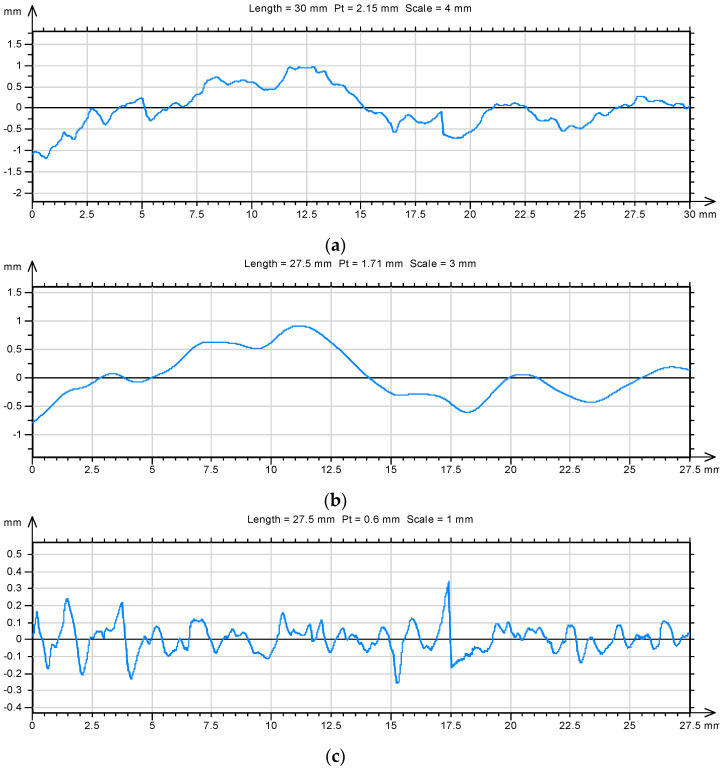
An example of profile line (**a**) separated out on the surface of a mortar sample modified with aluminum nanoxide in the amount of 1% after 7 days of maturation (*D* = 1.16) and its waviness profile (**b**) and roughness profile (**c**) (*D* = 1.17).

Additionally, one of the standard parameters in roughness analysis was determined as well as the total height of the *Pt* profile. Additionally, the so-called waviness and roughness profile was extracted from each profile line (Figure 9 and Figure 10).

In the case of the roughness profile, the fractal dimension *D* and the total height of the roughness profile were recalculated. The obtained results are summarized in Table 7 and Table 8.

A qualitative analysis (Snedecor–Fisher F test) of the impact of the mortar composition modifier used on the morphology of the fracture surface formed as a result of cracking was carried out and described by selected fractographic parameters. The obtained significance values *p* less than 0.05 proved a significant influence of the nano-Al_2_O_3_ addition on selected fractographic parameters.

The analysis showed a statistically significant effect of aluminum nanoxide on the value of the fractal dimension *D* and on the value of the fractal dimension of the *D_RP_* roughness profile according to a significance level of 0.05. At the same time, a differentiated effect of introducing nano-Al_2_O_3_ into the composition of mortars was shown on the second fractographic parameter, i.e., total height of *Pt* profile and total height of *Pt_RP_* roughness profile. The results of the analysis are presented in Table 9.

Based on the results of fractal studies, the influence of the applied modifier on the morphology of the mortar fracture surface formed as a result of destruction, characterized by profile lines separated out from these surfaces, was demonstrated (Table 7 and Table 9).

**Figure 11:**
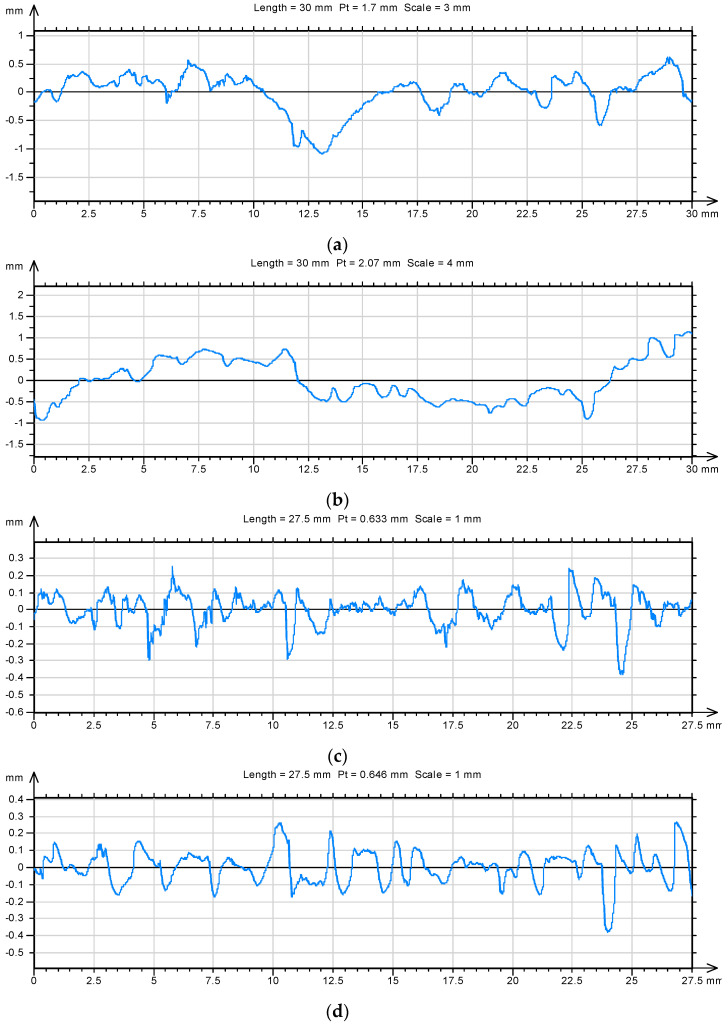
Summary of profile lines isolated on the surfaces of unmodified mortar samples: (**a**) (*D* = 1.22) and with 1% aluminum nanoxide (**b**) (*D* = 1.13) after 28 days of maturing and the corresponding roughness profiles (**c**) (*D* = 1.25) and (**d**) (*D* = 1.15).

The lowest values of fractal dimension *D* of profile lines of fracture surfaces were obtained for mortars modified with nano-Al_2_O_3_. The lower fractal dimension *D* indicates a reduction in the roughness on the fracture surface and, at the same time, a more homogeneous structure of the hardened cementitious composites. (Figure 11).

**Figure 12:**
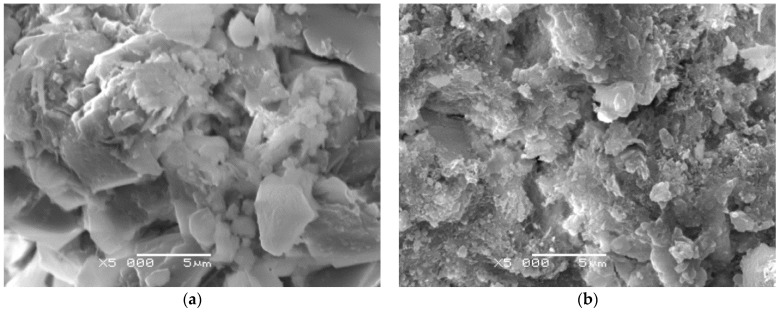
Visible microstructure of the cement paste (**a**) without modifier and (**b**) with 1% of aluminum nanoxide.

The results of the fractographic analyses also confirmed SEM observation with the use of a JEOL 5500 LV scanning microscope. Figure 12 shows images of the micro-structure of the cement paste representative for the given compositions. SEM tests were performed on the fracture surfaces obtained as a result of the destruction of the samples used for the crack resistance test. Observations of the microstructure of the hardened cement grout containing 1% aluminum nanoxide indicated lower porosity of this grout (Figure 9b) and thus less complexity of the fracture surface than in the case of the cement grout not modified with aluminum nanoparticle. The formation of small size crystals clusters in the C–S–H phase was noticeable in comparison with the unmodified slurry.

Mortars for which flatter fracture surfaces were obtained (smaller fractal dimension *D*) were also mortars with higher compressive strength. The lowest values of fractal roughness profile of *D_RP_* were also obtained for mortars modified with alumina nanoparticles (Table 7). The addition of aluminum nanoxide to the composition of the mortars, of much greater fragmentation than cement grains, resulted in filling the voids between the grains of hydrated cement with the pozzolanic reaction nano-Al_2_O_3_ products and with Ca(OH)_2_. This resulted in a greater homogeneity of the microstructure of the hardened mortar and a reduction of the fracture surface roughness. The values of both fractographic parameters (*D*, *D_RP_*) for mortars modified with aluminum nanoxide were about 10% lower than the values of the same fractographic parameters for unmodified mortars.

An in-depth statistical analysis (Table 10) of the effect of mortar age on the value of both fractal dimensions (*D*, *D_RP_*), with a significance level of 0.05 adopted, showed changes in the microstructure of the hardened cement paste for each series of mortars modified with aluminum nanoxide. The fractal dimension of the *D_RP_* roughness profile proved to be the most sensitive to changes. In this case, all the values of the significance level limit p were close to 0, which proves the influence of mortar age on the value of the fractal dimension of the *D_RP_* roughness profile.

The morphology of the fracture surface and its two-dimensional image (profile line) is closely related to the microstructure of the grout. Modification of the microstructure with aluminum nanoxide resulted in low values of the fractal dimension of the *D_RP_* roughness profile in the first periods of study (after 7 days of curing) (Table 7).

**Figure 13:**
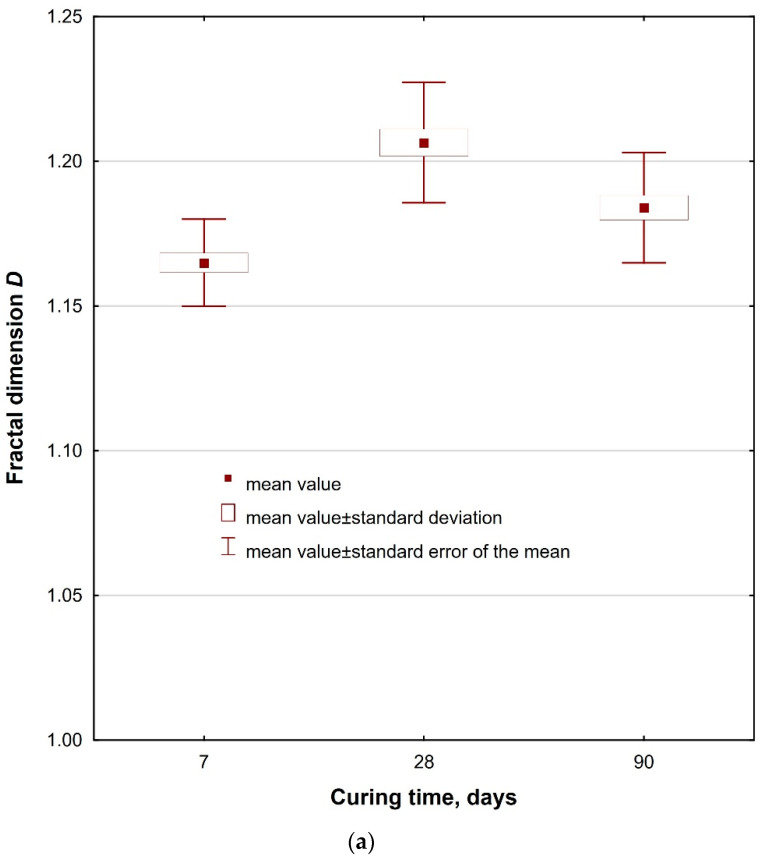

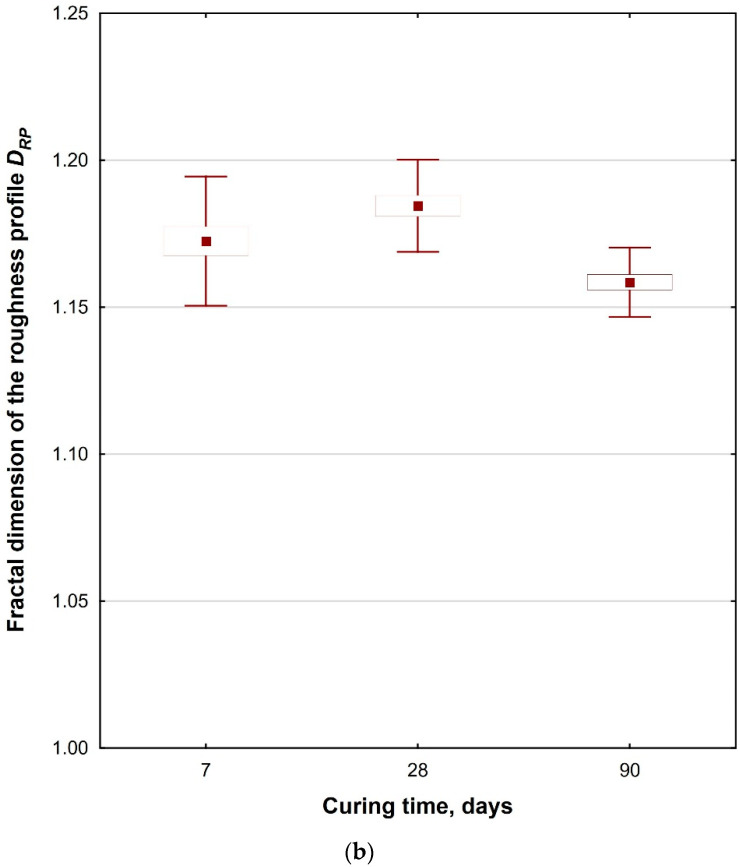
The influence of mortar age on (**a**) fractal dimension *D* and (**b**) fractal dimension *D_RP_* for MNA-1 mortars.

In the next periods, after 28 days of curing, the value of this dimension increased and then decreased again after 90 days. Probably, in the first period, the positive effect of the reaction of aluminum nanoxide with calcium hydroxide became visible, causing rapid sealing and strengthening of the grout microstructure. In the subsequent period, the cement reaction may have led to the formation of weaker hydration products, which are considered to be Ca(OH)_2_ crystals, which at the same time increased the dimension of the fractal roughness profile. The further process of cement hydration and building a structure based on dicalcium silicates (which provide greater strength than tricalcium silicates) increased the strength and reduced the fractal dimension of the *D_RP_* roughness profile (Table 7). The dimensional changes of the fractal roughness profile are shown in Figure 13.

Further statistical analysis showed the differentiation of the impact of introducing nano-Al_2_O_3_ into the mortar composition on the value of the total height of the *Pt* profile and the value of the total height of the *Pt_RP_* roughness profile (Table 9).

After 90 days of curing, the influence of the modifier on the value of the total height of the *Pt_RP_* roughness profile is shown. The total height of the *Pt_RP_* roughness profile of nano-Al_2_O_3_ modified mortars was approximately 32% lower than the total height of the *Pt_RP_* roughness profile of the control mortars (Table 8). This is probably related to the pozzolanic reactions involving nano-Al_2_O_3_ and the increase in the homogeneity of the microstructure of the hardened cement paste.

The conducted analyses confirmed greater usefulness of using fractal parameters (*D* fractal dimension) to describe the fracture surface morphology than the traditionally used fractographic parameters (*Pt* and *Pt_RP_*).

The fractal dimension, especially fractal dimension of roughness profile, turned out to be the parameter sensitive to modifications made in the composition of mortars, including changes related to curing time.

The statistical analysis of the results of roughness profile fractal dimension *D_RP_* included ANOVA analysis of the significance of the impact of effects, including the analysis of the equality of means and homogeneity of variance, performed for all the fifteen series of mortar (five series per each mortar age). The F Snedecor–Fisher test indicated a statistically significant impact of the variables (proportion of nanopowder and mortar age) on the roughness profile fractal dimension *D_RP_*, the critical level of significance *p* close to 0 (test statistic F = 53.44). Moreover, the homogeneity of variance was confirmed, and the critical level of significance level was achieved *p* = 0.06.

**Figure 14:**
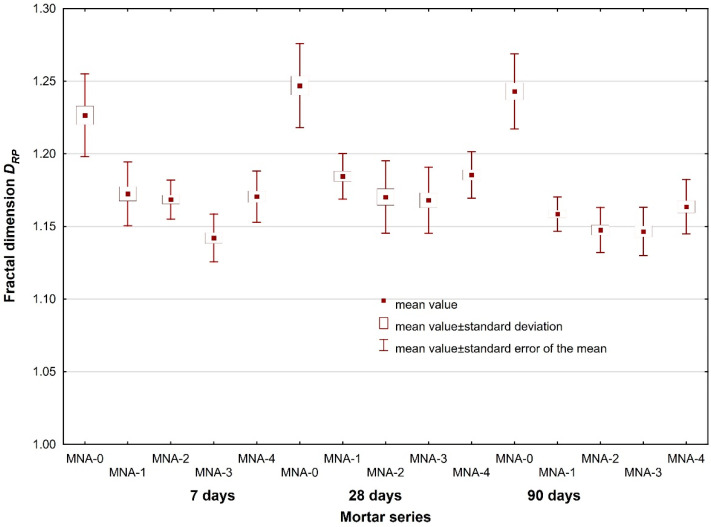
Scatter of fractal dimension *D_RP_* vs. nano-Al_2_O_3_ proportion and mortar age.

Once the variance homogeneity was achieved, it was possible to look for a description of the impact of the adopted variables (nanopowder proportion and mortar age) on the roughness profile fractal dimension *D_RP_* in the form of functional dependencies (Figure 14).

Employing the multiple regression method, adopting mortar age and nano-Al_2_O_3_ proportion as independent variables as well as for the adopted significance level, a dependence for the prediction of roughness profile fractal dimension *D_RP_* was obtained:

(5) $$D_{RP}=1.214-0.00011\cdot T-0.0302\cdot x,\;N\;=\;300,\;R\;=\;0.57,\;p\;=\;0.000$$

where *T* is the age of mortar, *x* is the nano-Al_2_O_3_ proportion and *N* is the number of cases.

Dependence (5) was obtained on the basis of 300 actual results of roughness profile fractal dimension determined following the profile lines separated out from the mortar fracture surfaces. The resulting correlation may be classified as good fitting of the results of actual physical experiments to the expected ones. The relative contribution of each of the independent variables (mortar age (T), nano-Al_2_O_3_ proportion (*x*)) to the prediction of the dependent variable (*D_RP_*) was compared. In this case, the nano-Al_2_O_3_ proportion was an over 5.6 times greater predictor of the roughness profile fractal dimension than mortar age. As follows from model (5), both mortar age and nano-Al_2_O_3_ proportion decreased the roughness profile fractal dimension. A lower profile roughness indicated a more homogeneous microstructure of hardened cement paste and better sand grains to paste binding.

Due to the character of the fracture surface of materials of cementitious matrix, the values of fractal dimension obtained for a single surface fracture were widely scattered. However, as shown in [50], and as the results of ANOVA analysis indicate, considering the results scatter connected with the accuracy of the methods employed, the differences in the values of roughness profile fractal dimension for concretes of different composition were statistically significant. This is an important practical conclusion as it confirms the applicability of this type of research and possibility of drawing conclusions based on the results, since it indicates sufficient sensitivity of the methods applied.

## 4. Conclusions

Based on the research, the following conclusions were drawn:

- The addition of aluminum nanoxide as a partial replacement for cement affects the consistency of mortars. As the content of nano-Al_2_O_3_ increases, so does the water demand for the binder. In order to prevent this, an admixture based on polycarboxyates was used, which, in addition to the function of a dispersing agent, acted as liquefier.
- Aluminum nanoxide has a positive effect on the early compressive strength of mortars but also on the compressive strength of mortars after 28 and 90 days of curing compared with the reference mortar samples. The highest compressive strength of mortars modified with nano-Al_2_O_3_ is demonstrated by the mortar with the addition of 1% alumina nanoparticles. Compared with the reference mortar (MNA-0 series), the MNA-1 series mortar shows an increase in compressive strength after 7 days by about 20%. With the increase of the addition of aluminum nanoxide, the compressive strength of the mortars decreases, but it is still higher than the compressive strength of the reference mortars. The decrease in compressive strength is caused by the formation of nano-Al_2_O_3_ agglomerates in the structure of mortars. This phenomenon results from the high specific surface area of the alumina nanoparticles.
- The results of the qualitative analysis (F Snedecer–Fisher test) proved a statistically significant impact of independent variables (nano-Al_2_O_3_ proportion and mortar age) on the output values (flexural and compressive strengths). The multiple regression method employed additionally enabled a description of the dependencies in the form of regression models (1) and (2). The results revealed that in model (1) the impact of the variable of mortar age on mortar flexural strength prediction is about three times greater than that of the variable of nano-Al_2_O_3_ proportion. In model (2) the impact of nano-Al_2_O_3_ proportion turned out to be negligible.
- For compressive strength, models (3) and (4), obtained by means of a non-linear estimation method, yielded better fitting of the results of empirical tests to the expected ones and indicated the significance of the impact of the variable nano-Al_2_O_3_ proportion. The performed analysis confirms a more complex impact of nano-Al_2_O_3_ proportion, in particular on mortar flexural and compressive strengths. The proposed models are applicable to mortar design.
- Alumina nanoparticles have a positive effect on the capillary adsorption coefficient. Compared with the reference mortar, the capillarity of mortars modified with nano-Al_2_O_3_ is lower by about 15%. The lowest capillary adsorption coefficient was demonstrated by the series of MNA-1 mortar with 1% content of aluminum nanoxide. Compared with the reference mortar, the capillary adsorption coefficient of MNA-1 mortar is lower by 21%. The lower capillarity of mortars modified with nano-Al_2_O_3_ is mainly due to the filling of capillary pores with aluminum nanoxide particles.
- The results of the mortar absorbability tests showed a minor effect of the addition of alumina nanoparticles on the absorbability of the tested cement mortars. All the results are comparable and oscillate within 8.6%.
- Fractographic analysis showed a significant effect of the modifier in the form of alumina nanoparticles on the value of fractal dimension *D* and fractal dimension of *D_RP_* roughness profile. The lowest values of fractal dimension *D* of profile lines of fracture surfaces were obtained for mortars modified with nano-Al_2_O_3_. Smaller fractal dimension *D* proves the reduction of unevenness on the fracture surface and, at the same time, a more homogeneous structure of the hardened cementitious composites. Mortars for which flatter fracture surfaces were obtained were also mortars with higher compressive strength. The fractographic parameters traditionally used to describe roughness were also found to be insensitive to changes in mortar structure. The authors suggest that the fractal dimension of the profile line should be determined on the basis of the roughness profile analysis. This approach eliminates distortions resulting from profile line analysis that includes the waviness profile. This is confirmed by analyses of the change in the fractal dimension of the roughness profile during curing.
- Based on the results of multiple regression analysis performed in the format of the roughness profile fractal dimension, adopting the mortar age and nano-Al_2_O_3_ proportion as variables and for the adopted significance level of 0.05, dependence (5) was obtained that enables roughness profile fractal dimension *D_RP_* to be predicted. On the basis of model (5), it was stated that both mortar age and nano-Al_2_O_3_ proportion decrease the roughness profile fractal dimension. Lower profile roughness manifests a more homogeneous microstructure of hardened cement mortar and better sand grains to cement paste binding, which also corresponds to a higher flexural and compressive strengths of these mortars.

## Figures and Tables

**Table 1:** Chemical properties of CEM I 42.5 R cement.

Chemical Properties	Requirements	Average Values
Loss on ignition (%)	max 5.00	2.95
Insoluble parts (%)	max 5.00	0.75
Sulphate content (as SO_3_) (%)	max 4.00	3.24
Chloride content (as Cl^−^ (%)	max 0.100	0.06
Alkali content (as Na_2_O_eq_) (%)	-	0.75

**Table 2:** Physical properties of 42.5 R cement.

Physical Properties	Requirements	Average Values
Start of setting time (min)	min 60	182
Compressive strength (MPa)	-	-
after 2 days	min 20.0	31.3
after 28 days	42.5−62.5	56.9
Volume change (mm)	max 10	0.7
Surface area	-	4187

**Table 3:** Physical properties of sand 0–2 mm.

Physical Properties	Values
Aggregate dimension	0/2
Granularity	G_F_85
Aggregate grain density	2.65 ± 0.05 (Mg/m³)
Petrographic composition	sedimentary rocks (sandstone, limestone—up to 1%)—less than 15% metamorphic rocks (quartzites)—over 85% igneous rocks—none

**Table 4:** Physical properties of nano-Al_2_O_3_.

Physical Properties	Values
Physical state	Solid, powder
Purity	99.99%
Surface area	25 m^2^/g
Initial boiling point	3000 °C
Relative density	4 g/cm^3^
Viscosity	Kinematic (40 °C): >0.205 cm^2^/s (>20.5 mm^2^/s)

**Table 5:** Composition of mortar mixes.

Sample Designation	Cement, g	Sand, g	Water, g	Nano-Al_2_O_3_, g	SP, % of the Cement Mass
MNA-0	450	1350	225	-	-
MNA-1	445			4.5	0.5
MNA-2	441			9	1
MNA-3	436.5			13.5	1.5
MNA-4	432			18	2

**Table 6:** Flexural strength and compressive strength of nano-Al_2_O_3_ particle blended cement mortars.

Sample Designation	Flexural Strength, MPa			Compressive Strength, MPa		
	7 Days	28 Days	90 Days	7 Days	28 Days	90 Days
MNA-0	6.1 ± 0.14	7.4 ± 0.33	7.8 ± 0.33	28.2 ± 0.30	36.9 ± 0.61	42.0 ± 0.65
MNA-1	6.1 ± 0.17	7.5 ± 0.33	8.2 ± 0.12	34.1 ± 1.14	39.5 ± 0.42	47.3 ± 1.17
MNA-2	6.0 ± 0.18	7.1 ± 0.33	8.0 ± 0.37	32.4 ± 0.51	40.0 ± 0.55	46.3 ± 0.73
MNA-3	5.7 ± 0.09	6.8 ± 0.33	7.7 ± 0.10	31.6 ± 0.5	39.6 ± 0.78	44.6 ± 0.59
MNA-4	5.6 ± 0.32	6.9 ± 0.33	7.2 ± 0.45	29.9 ± 1.28	36.8 ± 1.08	44.1 ± 1.07

**Table 7:** Results of the fractal analysis of the profile line (fractal dimension *D*) and the roughness profile extracted from it (fractal dimension of the roughness profile *D_RP_*).

Sample Designation	Fractal Dimension *D* ± Standard Error of the Mean			Fractal Dimension of the *D_RP_* Roughness Profile ± Standard Error of the Mean		
	7 Days	28 Days	90 Days	7 Days	28 Days	90 Days
MNA-0	1.23 ± 0.005	1.24 ± 0.004	1.23 ± 0.009	1.23 ± 0.006	1.25 ± 0.006	1.24 ± 0.006
MNA-1	1.17 ± 0.003	1.21 ± 0.005	1.18 ± 0.004	1.17 ± 0.005	1.18 ± 0.004	1.16 ± 0.003
MNA-2	1.18 ± 0.005	1.19 ± 0,003	1.19 ± 0.007	1.17 ± 0.003	1.17 ± 0.006	1.15 ± 0.003
MNA-3	1.15 ± 0.004	1.16 ± 0.005	1.16 ± 0.006	1.14 ± 0.004	1.17 ± 0.005	1.15 ± 0.004
MNA-4	1.16 ± 0.004	1.19 ± 0.005	1.18 ± 0.004	1.17 ± 0.004	1.19 ± 0.003	1.16 ± 0.004

**Table 8:** Results for the total height of the *Pt* profile and the roughness profile.

Sample Designation	The Greatest Total Height of the Profile *Pt* (mm) ± Standard Error of the Mean			The Highest Total Height of the *Pt_PR_* Roughness Profile (mm) ± Standard Error of the Mean		
	7 Days	28 Days	90 Days	7 Days	28 Days	90 Days
MNA-0	2.2 ± 0.12	1.9 ± 0.15	2.8 ± 0.27	0.8 ± 0.59	1.0 ± 0.16	1.0 ± 0.16
MNA-1	2.9 ± 0.93	2.0 ± 0.16	2.0 ± 0.09	0.7 ± 0.05	1.2 ± 0.21	0.7 ± 0.04
MNA-2	2.5 ± 0.26	1.5 ± 0.06	1.8 ± 0.10	0.92 ± 0.09	1.2 ± 0.35	0.7 ± 0.05
MNA-3	1.9 ± 0.14	2.2 ± 0.21	2.4 ± 1.50	0.8 ± 0.12	1.9 ± 0.15	0.7 ± 0.05
MNA-4	3.0 ± 0.16	1.9 ± 0.10	2.8 ± 0.20	0.9 ± 0.09	0.9 ± 0.08	0.7 ± 0.05

**Table 9:** Results of equality analysis of average fractographic parameters.

Fractographic Parameter	The Critical Significance Level *p* for Mortar After		
	7 Days	28 Days	90 Days
Fractal dimension *D*	close to 0	close to 0	close to 0
Fractal dimension of the roughness profile *D_RP_*	close to 0	close to 0	close to 0
Largest overall profile height *Pt*	0.34	0.008	0.099
Largest overall height of the roughness profile *Pt_PR_*	0.39	0.64	0.018

**Table 10:** The result of the statistical analysis of the equality of mean values of the fractal dimension (*D*, *D_RP_*) depending on the age of mortars.

Sample Designation	Critical Significance Level *p*	
	for the Fractal Dimension *D*	for the Fractal Dimension *D_RP_*
MNA-0	0.187	0.056
MNA-1	close to 0	close to 0
MNA-2	0.241	close to 0
MNA-3	0.300	close to 0
MNA-4	close to 0	close to 0

## Data Availability

Not applicable.
